# Triple sulfur-oxygen-strontium isotopes probabilistic geographic assignment of archaeological remains using a novel sulfur isoscape of western Europe

**DOI:** 10.1371/journal.pone.0250383

**Published:** 2021-05-05

**Authors:** Clément P. Bataille, Klervia Jaouen, Stefania Milano, Manuel Trost, Sven Steinbrenner, Éric Crubézy, Rozenn Colleter

**Affiliations:** 1 Department of Earth and Environmental Sciences, University of Ottawa, Ottawa, Canada; 2 Department of Biology, University of Ottawa, Ottawa, Canada; 3 CNRS, GET UMR 5563, Toulouse, France; 4 Department of Human Evolution, Max Planck Institute for Evolutionary Anthropology, Leipzig, Germany; 5 CNRS, CAGT, UMR 5288, Université Paul Sabatier, Toulouse, France; 6 INRAP (Institut National de Recherches Archéologiques Préventives), Cesson-Sévigné, France; University of Padova: Universita degli Studi di Padova, ITALY

## Abstract

Sulfur isotope composition of organic tissues is a commonly used tool for gathering information about provenance and diet in archaeology and paleoecology. However, the lack of maps predicting sulfur isotope variations on the landscape limits the possibility to use this isotopic system in quantitative geographic assignments. We compiled a database of 2,680 sulfur isotope analyses in the collagen of archaeological human and animal teeth from 221 individual locations across Western Europe. We used this isotopic compilation and remote sensing data to apply a multivariate machine-learning regression, and to predict sulfur isotope variations across Western Europe. The resulting model shows that sulfur isotope patterns are highly predictable, with 65% of sulfur isotope variations explained using only 4 variables representing marine sulfate deposition and local geological conditions. We used this novel sulfur isoscape and existing strontium and oxygen isoscapes of Western Europe to apply triple isotopes continuous-surface probabilistic geographic assignments to assess the origin of a series of teeth from local animals and humans from Brittany. We accurately and precisely constrained the origin of these individuals to limited regions of Brittany. This approach is broadly transferable to studies in archaeology and paleoecology as illustrated in a companion paper (Colleter et al. 2021).

## 1. Introduction

Isotopes have gained popularity for reconstructing the mobility of now-dead individuals or extinct animals in archaeology and paleoecology [1–5]. Isotopes are ubiquitous in organic tissues and vary predictably in the environment with biological and physical processes [6]. For several isotopic systems such as hydrogen, carbon or oxygen, the variations in isotopic abundances vary spatially, and these patterns are transmitted from inorganic (e.g., water, carbon dioxide) into organic tissues with some isotopic fractionation. As human and animal eat and drink, their organic tissues inherit an isotopic fingerprint from the local ecosystems that relate to the geographic location where the tissue was grown. In tissue with slow turnover rate (e.g., teeth, bones), the isotopic fingerprint is preserved post-mortem and is used to infer provenance or mobility of individuals. However, to obtain precise and unbiased provenance information, isotopes analysed in a tissue of interest needs to be compared to an isotopic baseline [7]. Without these baselines, isotope data from individuals can only be compared with each other or with existing databases [8]. While these points-to-points comparisons can be useful, they are spatially biased, limited, and often ambiguous [8]. A more quantitative approach to isotope provenancing is to use continuous-surface geographic assignment by comparing the measured isotope data in animal tissue to a map predicting isotope variations across the landscape, also called an isoscape [8]. This approach incorporates uncertainty and produces continuous-probability surfaces to visualize potential location of origin over an entire study area. Once generated, the probability surfaces from multiple isotopic systems are combined to summarize all provenance data into a probabilistic visual [9, 10]. Due to the lack of paleo-isoscapes, continuous-surface geographic assignments are rarely used in archaeology and paleoecology [5, 11, 12], hampering a shift towards more quantitative isotope provenancing in these fields [13].

Two isotopic systems, hydrogen/oxygen isotopes and strontium isotopes, have well-calibrated isoscapes, allowing continuous-surface assignment approaches in archaeology and paleoecology [13, 14]. The oxygen isotope composition (δ^18^O) of human and animal tissues that drink regularly (‘obligate drinkers’) mainly reflects the isotope composition of the local drinking water. Consequently, the isotopic composition measured in hard tissues (e.g., teeth enamel, bones) can be used to predict the isotopic composition of the water consumed by individuals using equations that account for metabolic isotopic fractionation [15–17]. These equations are specific to the mineral fraction analyzed (carbonate and phosphate) and the species considered. The oxygen isotope composition of the local precipitation, which controls the isotopic composition of local waters, is strongly influenced by climatic and geographic factors such as temperature, latitude, altitude and distance from the coast [18, 19]. These δ^18^O patterns in precipitation have been predicted at the global scale using existing isotopic data and geostatistical approaches [20, 21]. Strontium is predominantly transmitted to human tissues through ingested food [22, 23]. Strontium isotope ratios (^87^Sr/^86^Sr) are usually analyzed on teeth enamel because Sr is abundant in calcified tissues [23]. ^87^Sr/^86^Sr variations on the landscape vary at high resolution and are strongly influenced by the local geology. Unlike oxygen isotopes which fractionate with metabolic processes, the strontium isotope ratios are corrected for isotopic fractionation and only record mixing of isotopically distinct sources [13]. The isotope ratios are influenced by the age of the bedrock, the type of bedrock, its initial content in rubidium and the initial ^87^Sr/^86^Sr ratio at the time of the rock formation [24]. As rock ^87^Sr/^86^Sr is transmitted to ecosystems, many other sources of Sr with isotopically distinct ^87^Sr/^86^Sr values can influence the bioavailable ^87^Sr/^86^Sr ratios [13]. For example, the addition of aerosols, mixing occurring during soil pedogenesis, differential weathering rates of minerals, anthropogenic inputs or biological processes can influence the bioavailable ^87^Sr/^86^Sr values [25]. All those parameters cannot be easily predicted using a mechanistic modeling approach, but machine-learning models have been able to predict bioavailable ^87^Sr/^86^Sr variations with sufficient precision for isotope geolocation [5, 13, 26–28].

Sulfur isotopes provide provenance information but because of the lack of method to predict isotopic variations on the landscape, they have never been used in continuous-surface geographic assignments. Sulfur isotope composition (δ^34^S) in collagen is controlled by the isotopic composition of the food consumed [29, 30]. Plants uptake sulfur from two main sources: soil minerals and aerosols. Marine sulfur, either from sea salt or from dimethylsulfide (DMS), has a high δ^34^S value >15‰, and is transferred to terrestrial environments via sea spray, and aerosols [31–33]. Aerosols are the main source of plant sulfur close to the coast and in soils whose parent rock contains little sulfur [34]. Geological sulfur is weathered from bedrock and has a variable sulfur isotopic composition (-15 to 15‰) [34]. The contribution of bedrock as a sulfur source to ecosystems varies among localities, with regions dominated by S-rich rock (e.g., evaporites, sulfides) generally showing higher contribution from geological sources [34]. Sulfur sourced from sedimentary sulfides and igneous minerals has generally low δ^34^S values, whereas sulfur sourced from geological sulfates has high δ^34^S values [34]. The mixing of these isotopically distinct sources controls a large part of δ^34^S variations in ecosystems, whereas metabolic and trophic isotopic fractionation tend to be small [30, 31, 35–37]. In modern times, the human diet mixes products from multiple locations and sources, complicating the interpretation of δ^34^S patterns [38]. In archaeological times, however, most individuals lived in rural areas, eating and drinking mostly local food. Consequently, the δ^34^S values in their tissues primarily reflect that of local ecosystems. In archeological times, individuals from urban and affluent areas, also consumed dominantly local to regional food even though significant amount of exotic food sources might have also contributed to the diet as in modern times [38]. In Rennes, however, a limited amount of exotic food seemed to have to be imported and mainly consisted in wine from Bordeaux [39]. Individuals living in coastal areas characterized by a high deposition rate of marine sulfates, typically exhibit high δ^34^S values in their collagen. Individuals from inland areas have typically lower but more variable δ^34^S collagen values (-15 to 15‰), because the δ^34^S values of their food are less influenced by marine sulfates and more influenced by geological controls [38, 40]. One major exception to this pattern would be individuals who consume a high proportion of seafood with high δ^34^S values while leaving in inland regions, but, except for a few regions [41], this would have been a rare occurrence in archaeological times.

Here, we provide a complete framework to apply δ^34^S values for isotope-based, continuous-surface geographic assignment in archaeology and paleoecology. To enable continuous-surface geographic assignment using δ^34^S values, we first compiled 2,680 sulfur isotope analyses in collagen of archaeological human and animal teeth from 221 sites. We used this compilation to develop a locally calibrated human enamel δ^34^S isoscape for Western Europe using machine-learning regression. We used this isoscape to apply continuous-surface δ^34^S assignments and combined δ^34^S, ^87^Sr/^86^Srand δ^18^O into a triple-isotope geographic assignment framework. We used this framework to provide the most precise estimate of origin for a series teeth of local individuals recovered from a major medieval archaeological site in Rennes, France. We concluded this study by underlining how triple isotope continuous-surface geographic assignments provide an effective quantitative provenance tool in archaeological and paleoecological studies, as illustrated in a companion study [42].

## 2. Material and isotope methods

All statistical analyses conducted in this study used the R programming language version Rx64 3 4.2. (https://www.r-project.org/). All the scripts generated in this study and data required to run the scripts are available in the S1 and S2 Scripts.

### 2.1 Sulfur isoscape

#### 2.1.1 Data compilation

To characterize the δ^34^S baseline in human collagen across Western Europe, we compiled all the δ^34^S data published in human and animal collagen across Europe from the Mesolithic to the XXth century (S1 Dataset). During the Holocene, changes in the geology and climate of Western Europe were small, likely leading to stable δ^34^S patterns on the landscape [43]. We recorded the country, locality name, and species. We also compiled the latitude, longitude and reported the archeological period for each individual sample. In a few cases, geographic coordinates were reported by the authors in the publication. Geographical coordinates of numerous sites were found on GeoHack and, for a few occasions, on Wikipedia. When these were not included, we used Google Earth to georeference reported geographic information (e.g., maps or locality names). When necessary, authors of publications were contacted to clarify locality information. The distance to the coast was then obtained using the NASA dataset (https://oceancolor.gsfc.nasa.gov/docs/distfromcoast/) or, for Great Britain, the Doogal website (https://www.doogal.co.uk/DistanceToSea.php). We then screened all the δ^34^S data from poorly preserved collagen based on the following criteria C/S<300 and N/S<100 [44]. When only S data were available, we did not screen data. We also screened a few datasets for which diagenesis or contamination is explicitly mentioned in the publication (e.g., [45]). We also eliminated individuals that were explicitly designated as non-local in publication based on archeological evidences (i.e., we define non-locals as individuals coming from a distant locality >100 km). We did not consider data from aquatic animals or fish consumers to minimize dietary effect on δ^34^S variations. After screening, we calculated the average δ^34^S value for all individuals.

#### 2.1.2. Auxiliary variables

We assembled data on selected covariates that represent the main factors that impact variability in δ^34^S values. The variables include geology, climate (e.g., precipitation, temperature), soil proprieties (e.g., pH, clay content, organic matter), aerosol deposition (e.g., sea salt) and distance to the coast (Table 1). We resampled and reprojected all the selected environmental geospatial products into WGS84-Eckert IV 1km^2^ resolution and used latitude and longitude of each sampling site to extract the local values for each raster. We assumed that, in archaeological times, humans and animals mostly consumed local food (within 20 km), and their δ^34^S values likely reflected that of local food systems.

**Table 1:** List of geological, climatic, environmental and anthropogenic variables used in the multivariate regression.

Variables	Description	Resolution	Type	Source
**r.maxage_geol**	GLiM age attribute (Myrs)	1 km	D	[13, 45]
**r.minage_geol**	GLiM age attribute (Myrs)	1 km	D	[13, 45]
**r.meanage_geol**	GLiM age attribute (Myrs)	1 km	D	[13, 45]
**r.age**	Terrane age attribute (Myrs)	1 km	D	[46]
**r.ssaw**	Multi-models average sea salt wet deposition (kg.ha^-1^.yr^-1^)	1°×1°	C	[47]
**r.ssa**	Multi-models average Sea salt wet+dry deposition (kg.ha^-1^.yr^-1^)	1°×1°	C	[47]
**r.dust**	Multi-models average (g.m^-2^.yr^-1^)	1°×1°	C	[48]
**r.elevation**	SRTM (m)	90 m	C	[49]
**r.cec**	Cation Exchange Capacity	250 m	C	[50]
**r.ph**	Soil pH in H_2_O solution (x10)	250 m	C	[50]
**r.phkcl**	Soil pH in KCl solution (x10)	250 m	C	[50]
**r.clay**	Clay (weight %)	250 m	C	[50]
**r.orc**	Soil organic carbon (weight %)	250 m	C	[50]
**r.bulk**	Bulk density (kg m^−3^)	250 m	C	[50]
**r.bouguer**	WGM2012_Bouguer (mGal)	2 min	C	[51]
**r.map**	Mean annual precipitation (mm.yr-^1^)	30-arc sec	C	[52]
**r.mat**	*Mean annual temperature (°C)*	30-arc sec	C	[52]
**r.distance**	*Distance to the coast (km)*	30-arc sec	C	This study

D: Discrete; C: Continuous; GLiM: Global Lithological Map; CCSM.3: CommunityClimate System Model 3; SRTM: Shuttle Radar Topography Mission.

#### 2.1.3 Machine-learning multivariate regression

We combined the δ^34^S compilation and the extracted covariate into a regression matrix. We then tested two main approaches to predict δ^34^S variations across the landscape using the *GSIF* package in R [46]. Briefly, the *GSIF* package automatically fits multivariate regression-kriging model for a given set of points and covariates. It first fits a regression model (e.g. Generalized Linear Model, random forest model) and then fits variogram for the residuals. We tested the potential of generalized linear model and random forest model to predict δ^34^S variations. For conciseness, we only describe the results obtained from random forest regression kriging, which performed better than other models. The R script for applying this modelling approach, including the generalized linear model, is available in S1 Script. Random forest is a machine-learning algorithm trained by bootstrap sampling and random feature selection. Random forest creates multiple decision trees on different data samples where sampling is done with replacement to prevent overfitting. To make fair use of all potential predictors, the number of features split at each node of a tree is limited to some user-defined threshold. Ultimately, random forest aggregates the results of these decision trees to predict the mean value of the response variable. In our approach, the models were optimized using root mean squared error (RMSE) as the primary metric and a 10-fold repeated cross-validation scheme with 5 repetitions using 80% of the data for training at each iteration. To maximize model performance while minimizing the number of predictors included, we used the Variable Selection Under Random Forest (*VSURF*) package [47], which helps eliminate irrelevant and redundant variables. *VSURF* uses a two step-process, first ranking variables and then selectively adding variables into a model to minimize out-of-bag error. Once a model is optimized, we used variable importance purity measure and partial dependence plots to describe the relationships between the selected predictors and predicted δ^34^S. Ultimately, the δ^34^S isoscape was generated using the best performing random forest regression model.

### 2.2 Archaeological samples and study site

#### 2.2.1 Sample selection

For this study, we applied triple isotope geographic assignments using animal and human remains from a well-studied archaeological site in Brittany: the Dominican convent of Rennes [48, 49]. We selected this location because two archaeological mass graves were recently discovered at the site with dozens of individuals of unknown origin. The goal of this study is not to investigate these mass graves but to demonstrate the efficiency of using triple isotope geographic assignments for local animals and individuals. Individuals from the mass graves are investigated using isotope data in a companion paper (see [42] for details).

We selected a series of teeth from local archaeological fauna and humans recovered from graves within the Convent (Tables 2 and 3) and previously studied [50, 51]. The animal teeth were recovered from the hospital midden (XIII-XVIth) located on the same square of the convent, since no contemporaneous terrestrial fauna remains could be found on the site of the graves [51]. Historical resources document that pigs were raised within the city walls, whereas the cattle were coming from the countryside [52]. The dogs from this midden were assumed to belong to the people from the hospital and/or urban stray dogs [53]. We also sampled two local humans whose life has been documented: Louise de Quengo and Louis de Plessis. The first grew up and lived between Rennes and St Hernin (central Brittany, where her heart was probably buried with her husband’s body in 1656) [54]. The latter is a local noble who probably spent his life in Rennes or in the Morbihan region, also located in Brittany [48]. The teeth of these local individuals were sampled and analyzed for δ^18^O and ^87^Sr/^86^Sr. The dental enamel was first mechanically cleaned using a microdrill under a laminar flow box. Eight mg of dental enamel powder was then collected for δ^18^O whereas a chunk of enamel (10–15 mg) was taken for ^87^Sr/^86^Sr using a microsaw, at the same height of the tooth. We therefore analyzed both isotope systems on bioapatite of teeth enamel. We analyzed δ^18^O on the whole tooth enamel (also called structural carbonate) to ensure that the δ^18^O and ^87^Sr/^86^Sr analyses were conducted on the same substrate. While δ^18^O on structural carbonate is usually less resistant to diagenesis, there is a strong correlation between the δ^18^O of carbonates and phosphates in bioapatite [15, 55]. For δ^34^S analysis, we extracted the tooth root for collagen following the well-established collagen extraction protocol [56]. Some of the ^87^Sr/^86^Sr and δ^34^S values were published in previous studies and the methods of sample preparation and analysis are available in the corresponding publications [50, 51]. This sampling methodology insured that all three isotopes were analysed on tissue reflecting similar age allowing for triple isotope provenance.

**Table 2:** Nitrogen, carbon and sulfur isotope values in tooth collagen.

ID	Tooth	%C	%N	%S	C/S	C/N	δ^15^N	SD	δ^13^C	SD	δ^34^S	SD	Ref
Dog	P4	42.1	15.5	0.14	300	3.2	11.0	0.03	-19.6	0.03	13.3	0.14	[58]
Cow	M1	43.4	15.8	0.24	180	3.2	8.0	0.08	-21.7	0.08	12.0	0.04	[58]
Veal	?	42.8	15.6	0.17	251	3.2	8.6	0.03	-22.0	0.03	11.4	0.13	[58]
Sheep	?	42.4	15.5	0.14	302	3.2	9.6	0.02	-21.7	0.04	12.0	0.57	[58]
L. de Quengo	PM	42.32	15.38	0.26	162.7	3.3	14.1	-	-19.1	-	14.7	-	[58]
L. du Plessis	PM	43.67	15.89	0.29	150.6	3.2	14.0	-	-19.6	-	14.8	0.23	[58, 67]

Most data were collected in this study, except for a few individuals for which the reference is provided. C/S, N/S and C/N ratios are provided to underline the good preservation of the collagen from these samples. Delta values are expressed in ‰.

**Table 3:** Strontium, carbon and oxygen isotope values obtained in dental enamel.

ID	Tooth	%C	δ^13^C	δ^18^O	Ref	^87^Sr/^86^Sr	Ref
Dog	P4	5.2	-11.9	-5.0	This study	0.71270	[58]
Pig	P4	6.1	-9.5	-4.5	This study	0.71164	[58]
Cow	M1	6.4	-13.1	-6.0	This study	0.71467	[58]
Veal	?					0.71367	[58]
	P3	6.4	-11.4	-5.0	This study		
	P4	6.9	-11.1	-4.6	This study		
Sheep	?					0.71242	[58]
	M1	6.8	-14.2	-3.8	This study		
	M3	6.5	-13.2	-4.8	This study		
L. de Quengo	PM	5.43	-12.9	-4.0	This study	0.71079	This study
L. du Plessis	PM	4.74	-13.6	-3.0	This study	0.71246	This study

Most data were collected in this study, except for a few individuals for which the reference is provided. Typical SD (according to standards) were 0.01–0.12 permil for carbon isotope ratios, 0.03–0.10 permil for oxygen isotopes, and 0.00005 for Sr isotope ratios.

#### 2.2.2 Sample preparation and isotope analyses

The column chromatography for Sr purification was modified from Deniel and Pin [57]. The Sr isotope analyses were performed in the Department of Human Evolution, Max Planck Institute of Evolutionary Anthropology (MPI-EVA) as well as in the University of Calgary, Thermo Scientific^TM^ Neptune^TM^ high-resolution multi-collector inductively coupled plasma mass spectrometer (MC-ICP-MS; Thermo Fisher Scientific, Bremen, Germany). The following cup configuration was used: 5 Faraday cups (88Sr:H4, 87Sr+87Rb:H2, 86Sr+86Kr:C, 85Rb:L1, 84Sr+84Kr:L2). The flow rates were of 15L/min for the cool gas, 0.8 L/min for the auxiliary gas and about 1.2 L/min for the sample gas. Sample and skimmer X Ni cones were used. The long-term reproducibility of the ^87^Sr/^86^Sr measurement for NIST SRM987 is 0.71025 ± 0.000024 (1 SD, n = 137). The long-term reproducibility of the ^87^Sr/^86^Sr measurement for NIST SRM 1486 (bone Meal) is 0.709299 ± 0.000026 (1 SD, *n* = 137).

Sulfur was extracted at the Department of Human Evolution of the MPI-EVA using the protocol of Talamo and Richards [56], and isotope analyses were performed by the company Isoanalytical Ltd. (Sandbach, UK). The δ^34^S analyses values are corrected for oxygen-18 contribution to the SO_2_ produced from each sample, and control samples are analysed alongside to verify the accuracy of the correction procedure. The samples and standards were weighted into tin capsules, loaded onto an EA-IRMS (ANCA-GSL/20-20, Europa Scientific, Crewe, UK). The ^34^S/^32^S ratios were derived by monitoring of *m/z* 48, 49 and 50 of SO^+^ produced from SO_2_. Standards used for calibration were NBS 127 (barium sulfate, δ^34^S = +20.3‰ vs. CDT, IAEA, Vienna, Austria). NBS 127, IAEA-S-1 (silver sulfide, δ^34^S = -0.3‰ vs. CDT, IAEA, Vienna, Austria) and IA-R025 (barium sulfate, δ^34^S = +8.5‰ vs. CDT, Iso-Analytical, Sandbach, UK). In addition, the standard NBS-1577A (powdered bovine liver, δ^34^S = 7.9‰ vs. CDT, NIST, Gaithersburg, USA) was used as a control. All δ^34^S values were reported to the international scale VCDT. Oxygen and carbon isotope ratios were also analyzed by the company Isonalytical Ltd. (UK). For δ^18^O and δ^13^C analysis, the samples and isotope standards were loaded onto a CF-IRMS isotope ratio mass spectrometer (ANCA-G/20-20, Europa Scientific, Crewe, UK). The isotope ratios were derived by monitoring of m/z 44, 45 and 46 of CO_2_ produced by phosphoric acid reaction in Exetainers (Labco, Lampeter, UK). Samples were measured directly against a carbonate reference material IA-R022 (Iso-Analytical working standard calcium carbonate, δ^13^C V-PDB = -28.63‰ and δ^18^O V-PDB PDB = -22.69‰) in order to account for temperature dependent isotope fractionation. Internal standards used for calibration were NBS18 (δ^13^C V-PDB, -5.01‰, SD = 0.05‰; δ^18^O V-PDB, -23.2‰, SD = 0.07‰) and IA-R066 (chalk, δ^13^C V-PDB = +2.33‰ and = -1.52‰).

### 2.3 Isotope geolocation

#### 2.3.1 Continuous-surface probabilistic assignment

In this study, we applied isotope geolocation using teeth isotopic data to assess the origin of the studied individuals during tooth formation (i.e., childhood to early teenager) using ^87^Sr/^86^Sr, δ^18^O and δ^34^S values. We applied the well-established continuous-surface assignment framework to determine the probability of origin of individuals by comparing the isotopic composition of the individual teeth with that of the corresponding isoscape [9, 10]. Using this framework, the most likely origin for a given sample x* for which δ^18^O, ^87^Sr/^86^Sr and δ^34^S have been analysed can be determined by evaluating the likelihood at each cell c of the isoscape, knowing the predicted isotopic mean (μ_c_) and standard deviation (σ) at each cell. Given the observed values of each isotope variable, the univariate standard normal distribution is used for calculating likelihoods. The standardization of the normal distribution is given by:

$$z^{*}=\frac{x^{*}-\mu_{c}}{\sigma_{c}}$$ (Eq 1)

The comparison of observed and predicted isotopic values allows evaluating the probability of origin of the sample at each location relative to all other locations using the normal probability density function as follows:

$${f(z}^{*}|c)=(\frac{1}{\surd 2\pi })exp\left[-\frac{z^{*2}}{2}\right]$$ (Eq 2)

where f(z*|c) is the probability that any given cell on the study area represented a potential origin for an individual origin z* knowing the S, Sr or O isotopic value in the tooth.

In other words, when comparing an observed isotopic value to a series of pixels on an isoscape, the probability of origin of a sample will increase when the observed isotopic value is close to the predicted isotopic value and vice versa. The value of the probability of origin will also be influenced by the uncertainty of the isoscape at each pixel. The equation above is valid when using single isotopic system geographic assignment. Under the assumption of independence between isotopic systems, the combined probability density is simply the product of the single isotope probability surfaces obtained from the equation above. The script to generate the assignments for teeth is available in S2 Script.

#### 2.3.2 Oxygen isoscape

δ^18^O variations in animal tissues are primarily inherited from drinking waters which follow primarily precipitation patterns [58]. The best approach to generate a well-calibrated δ^18^O with spatially-explicit uncertainty is to use the framework described in the *assignR* package [10]. In this package, data from known-origin are used to calibrate a relationship between modeled precipitation δ^18^O values and that of the tissue of interest. However, there is little data of known origin for δ^18^O of structural carbonates of bioapatite. Consequently, we developed δ^18^O isoscape for structural carbonates in a three-step process:

**Step 1:** We used existing prediction of δ^18^O variations in precipitation across Europe (mean and standard deviation).
**Step 2:** We converted the δ^18^O_c_ (δ^18^O of structural carbonate) into the more commonly analysed δ^18^O_p_ (δ^18^O of phospate).
**Step 3:** We converted the δ^18^O_p_ (δ^18^O of phospate) into δ^18^O_w_ (δ^18^O of drinking water).
**Step 1:** We used as a basis the regionalized cluster-based water isotope prediction (RCWIP) annual amount-weighted mean δ^18^O isoscape in precipitation and its associated uncertainty [20]. Both of these layers, mean and uncertainty, were downloaded and cropped to our study area.
**Step 2:** The precipitation water δ^18^O isoscape was then converted to a spatially explicit tooth carbonate isoscape. We first converted δ^18^O in carbonates to δ^18^O in phosphates using the equation in Iacumin et al. [55].

$$\delta^{18}O_{p}=\left(\delta^{18}O_{c}‐8.79\left(\pm 0.79\right)\right)/1.015\left(\pm 0.043\right)$$ (Eq 3)

We used this equation and not the more recent Chenery et al. [15] equation because the data used to calibrate the Eq 3 in Iacumin et al. encompassed our study area. δ^18^O in phosphates and carbonates of teeth are usually strongly correlated but phosphates are more commonly due to their resistance to diagenesis [55]. Consequently, there are many equations converting δ^18^O in phosphates to that of drinking water and we used the equation of Hoppe et al. [17].

$$\delta^{18}O_{w}=\left(\delta^{18}O_{p}‐21.28\left(\pm 0.51\right)\right)/0.68\left(\pm 0.04\right)$$ (Eq 4)

Together these two equations have an uncertainty of approximately 1‰ as calculated in Chenery et al. [15]. This uncertainty is added to the uncertainty of the precipitation isoscape used in step 1 [20].

By using this isoscape, we make several assumptions. First, we assumed the δ^18^O_w_ in Europe today are similar to those that occurred at the time these individuals lived ~500-years ago. This assumption is probably reasonable as atmospheric circulation, sea-level, temperature and topography were relatively similar to modern [43]. Even in cases where climate would be different than modern times (e.g., Last Glacial Maximum), maps of δ^18^O values in precipitation can be generated [59] and calibrated using the assignR package [10]. Second, climate variations occurring during the period of teeth formation did not impact their δ^18^O_c_. Third, the water the individuals drank was local and not coming from distant rivers coming from high altitudes or from evaporated lakes. Fourth, we assumed that the δ^18^O of the local food and water consumed by humans was not influenced by human cooking practices (e.g., boiling, brewing and cooking).

#### 2.3.3 Strontium isoscape

To model ^87^Sr/^86^Sr variations in human teeth, we used a published model that predicts ^87^Sr/^86^Sr ratio in bioavailable Sr across the world and its associated uncertainty [13]. Briefly, this model uses a multivariate random forest regression framework, coupling biogeoenvironmental covariates and bioavailable ^87^Sr/^86^Sr data, to predict the average ^87^Sr/^86^Sr at each pixel and its associated uncertainty (1 standard deviation). The model explained more than 60% of the variance after cross-validation over a bioavailable ^87^Sr/^86^Sr dataset across Western Europe. When applying this isoscape built and calibrated on modern data, we make the assumption that the present day bioavailable ^87^Sr/^86^Sr ratio is a good estimate of the bioavailable Sr available to the individuals studied here ~530-years ago. As most of the bioavailable ^87^Sr/^86^Sr data used to calibrate the model are from undisturbed areas without farming activities, we assumed that fertilizers had little impact on the predicted ^87^Sr/^86^Sr variability [13]. In addition, the geology, climate and aerosol deposition of France is believed to have changed little in the last few centuries, suggesting that bioavailable ^87^Sr/^86^Sr variability was very similar to present-day [60].

## 3. Results

### 3.1 Sulfur isotope

The screened dataset includes 187 averaged values from individual locations published between 2001 and 2020 and are given with the associated references in S1 Dataset. The δ^34^S values are normally distributed (Shapiro Test, p-value<0.05). δ^34^S values range from -5.1‰ to 21.2‰ and average 8.29‰. Standard deviations for each site are ranging from 0.07 to 5.3‰ and average 1.77‰. Some geographical regions of Western Europe are underrepresented due to the lack of publications on S isotopes in bone collagen: Central Spain, Northern Germany, Denmark and Ireland.

**Fig 1:**
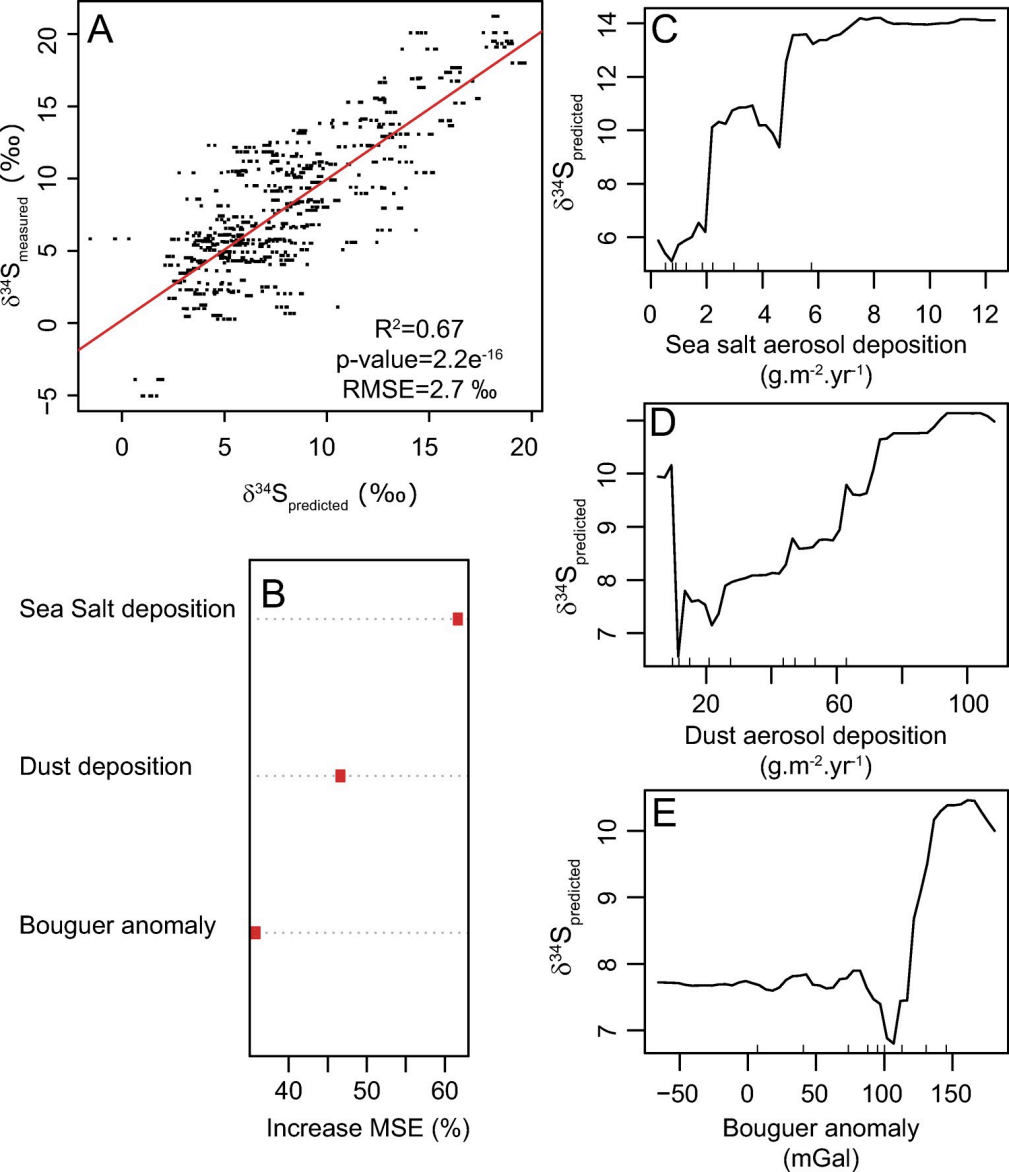
n-fold cross-validation and predictor for the δ^34^S random forest regression model. **A:** 5-fold cross-validation between predicted and measured δ^34^S values. RMSE = Root Mean Square Error. The red line is the best-fit linear model. **B:** Variable importance plot showing the increase in mean squared error predictions (estimated with out-of-bag-CV) as a result of the variable being permuted. Higher importance values indicate more weight as a predictor. **C-E:** Partial dependence plots showing the relation between each selected predictor on the predicted δ^34^S values. The R script to generate these figures is available in S1 Script.

δ^34^S values in the compiled database displayed a normal distribution. After *VSURF* feature selection, sea salt aerosol deposition, dust aerosol deposition (i.e., the deposition of mineral dust primarily generated in arid regions and transported by atmospheric circulation), and Bouguer anomaly (i.e., The remaining value of gravitational attraction after accounting for the theoretical gravitational attraction at the point of measurement which is influenced by geology and topography) were the dominant predictors of the δ^34^S values (Fig 1B). The resulting model performed well, explaining close to 67% of the variance with a Root Mean Square Error (RMSE) inferior to 2.8‰ (Fig 1A). The value of 2.8‰ represents ~10% of the full range of measured δ^34^S values over the compiled dataset. This uncertainty is relatively uniform across the prediction range (i.e., residuals are normally distributed). We used partial dependence plots to investigate the relationship between predicted δ^34^S values and *VSURF-*selected predictors (Fig 1C–1E).

**Fig 2:**
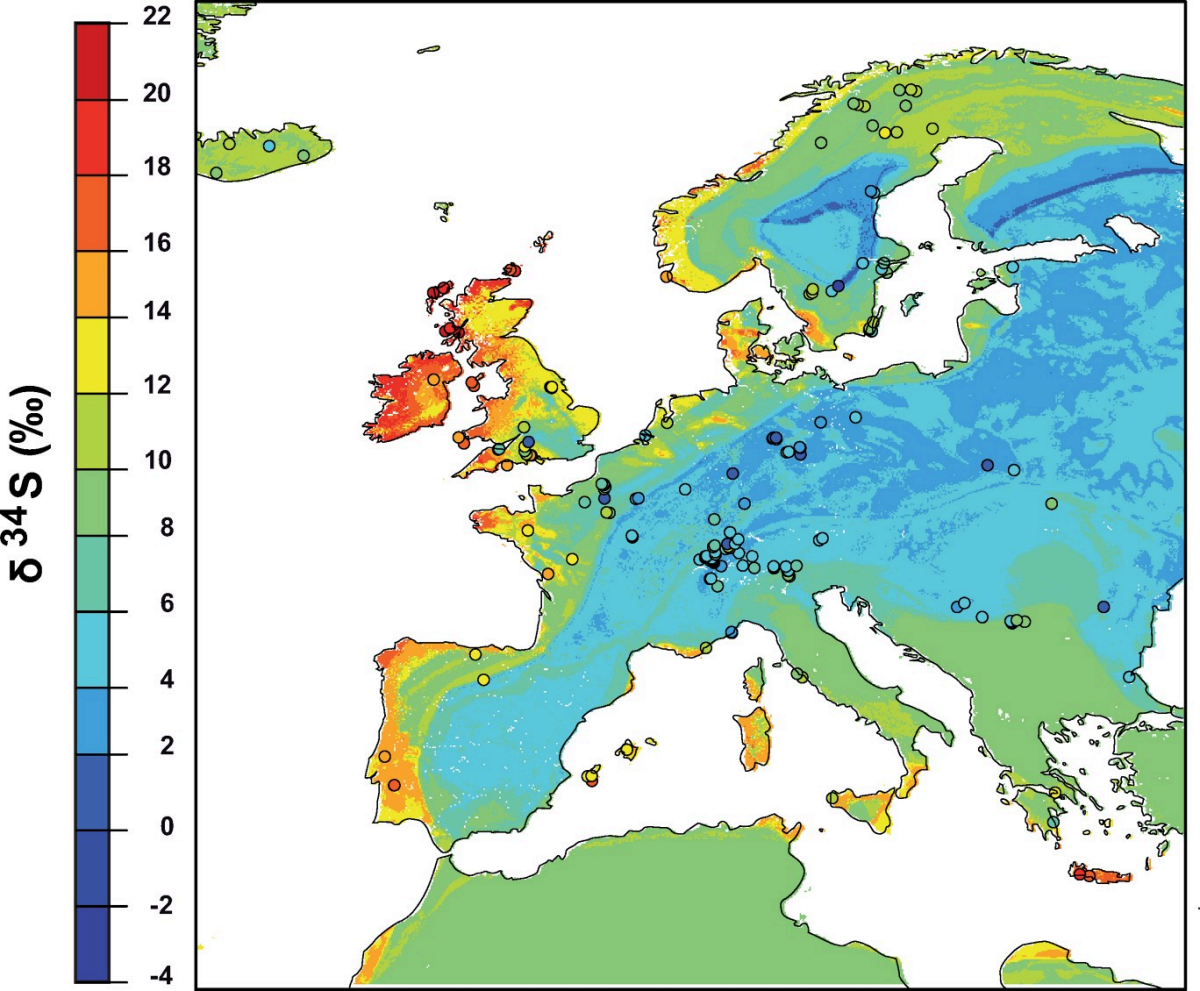
Sulfur isoscape of human remains across Europe. Spatial distribution of the sulfur isotope composition (δ^34^S) across Europe from a compilation of animal and human teeth from post-Mesolithic times (S1 Dataset) and isoscape developed in this study.

The random forest regression model produced a δ^34^S isoscape in human teeth that displays strong spatial patterns associated with aerosol deposition and geology. Sites located in coastal Western Europe have higher δ^34^S values (Fig 2). The highest values are found on the West coast of England and Scotland. Conversely, sites located in interior regions of France, Germany and the Scandinavian Peninsula all have lower δ^34^S values. The δ^34^S isoscape follows a similar pattern with high δ^34^S values on the coast and lower δ^34^S values inland.

We used the δ^34^S isoscape (Fig 2A) to apply probabilistic geographic assignments as described earlier. However, while random forest provides a mean δ^34^S prediction using the selected predictors, there are no built-in features to assess spatially explicit model uncertainty. To circumvent this issue, we assumed that uncertainty of the δ^34^S isoscape was constant and equal to the RMSE of 2.8‰.

### 3.2 Isotopic data from local individuals

The δ^34^S, δ^18^O and ^87^Sr/^86^Sr ratios of the local animals and humans show a tight distribution (Tables 2 and 3). δ^34^S values of animals cluster around 12‰, whereas humans show slightly higher values around 14‰. Most individuals have δ^18^O values ranging between -5‰ to -4‰ except for a cow with lower δ^18^O values at -6‰ and Louis du Plessis with higher δ^18^O values at -3‰. Except for Louise de Quengo, most individuals have ^87^Sr/^86^Srratios that fall between 0.712 and 0.713.

### 3.3 Continuous-surface geographic assignment

We compared the precision of single, dual, and triple isotope geographic assignments. We defined precision as the ability of the model to restrict the area of origin represented by the zone with high probability within the study area. We arbitrarily defined zones of high probability using as a threshold the highest 33.3% of the probability distribution as done in past studies [10, 61]. We created binary surfaces with all cells with low probabilities receiving zero and all cells with high probabilities receiving 1. These binary surfaces were then used to calculate the area of highly probable origin relative to the total study area.

Assuming all the individuals were local, the isotopic systems showed high accuracy for ^87^Sr/^86^Sr and δ^34^S assignments. For both δ^34^S and ^87^Sr/^86^Sr assignments, all individuals had Rennes or its immediate surrounding region (within 10 km) as an area of high probabilities (as defined above). The δ^18^O values, however, were not consistently accurate. The cow and Louis du Plessis showed δ^18^O values outside the areas of high probabilities (as defined above). This does not indicate that these individuals are incompatible with Brittany but that their δ^18^O values is not diagnostic of this region.

While most single isotope assignments are accurate, they showed low precision relative to dual and triple isotope assignments. At high probability (i.e., most probable 33.3%), δ^18^O assignment removed on average 82.1% of the study area, ^87^Sr/^86^Sr assignment removed 94.7% and δ^34^S assignment removed 93.6%. Dual ^87^Sr/^86^Sr -δ^18^O assignment removed 95.5%, dual ^87^Sr/^86^Sr—δ^34^S removed 98.3% and dual δ^18^O - δ^34^S removed 93.6%. Lastly, triple assignment removed 98.4%.

**Fig 3:**
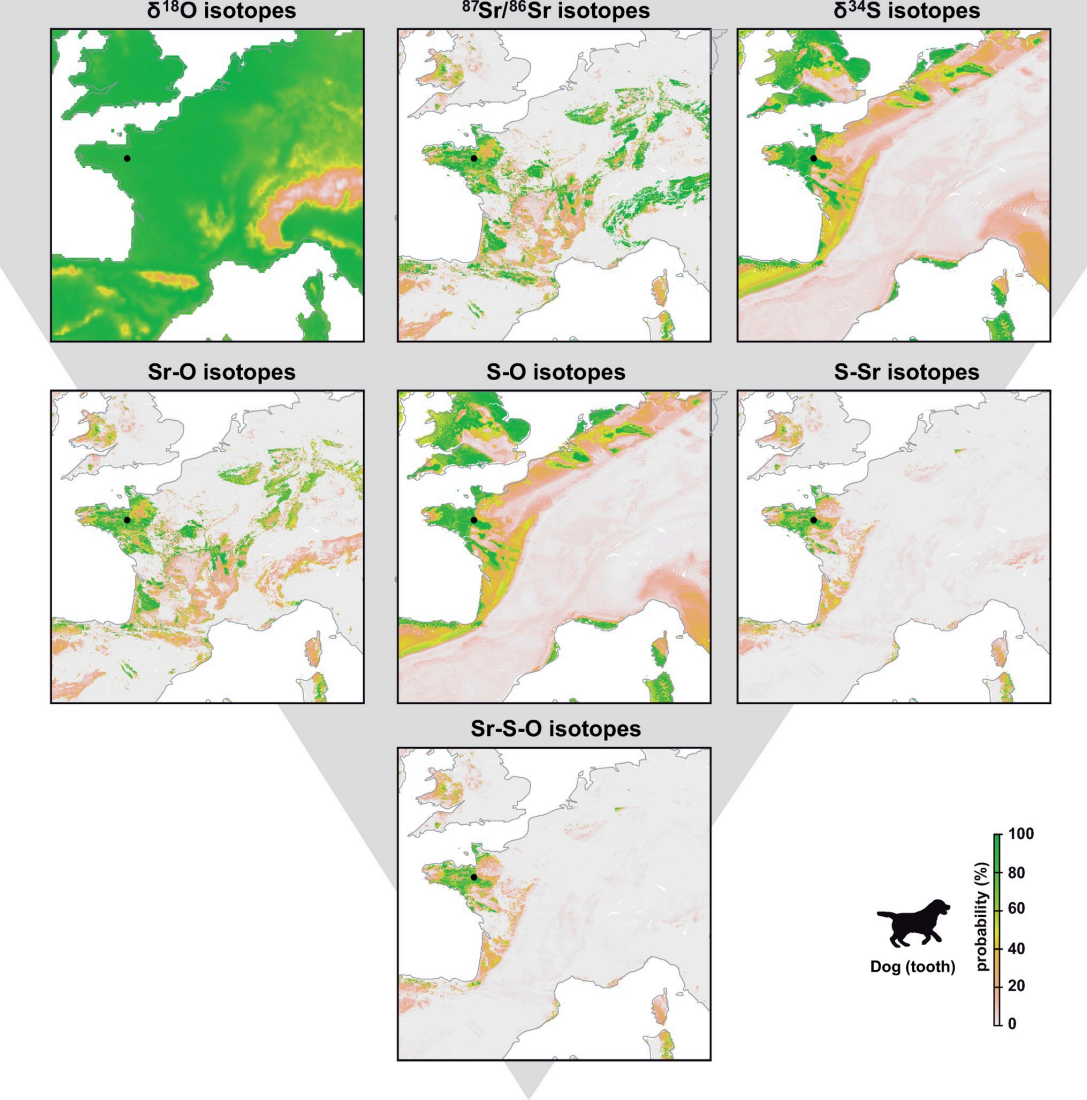
Maps showing the probability of tooth origin of the dog according to the selected isotopes (strontium, sulfur and/or oxygen). Depending on the isotopes and combinations of isotopes used, the geographical area of assignment is increasingly more precise. To generate these figures the script is available in S1 and S2 Scripts.

**Fig 4:**
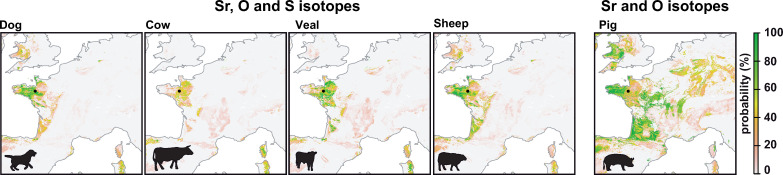
Maps showing the probability of geographic origins of the animals based on strontium, sulfur and oxygen isotope data in teeth. For the sheep, strontium and oxygen isotope values represent an average of two teeth M1 and M3. Sulfur isotopes were not analysed for the pig individual due to budget constraints. To generate these figures the script is available in S1 and S2 Scripts.

**Fig 5:**
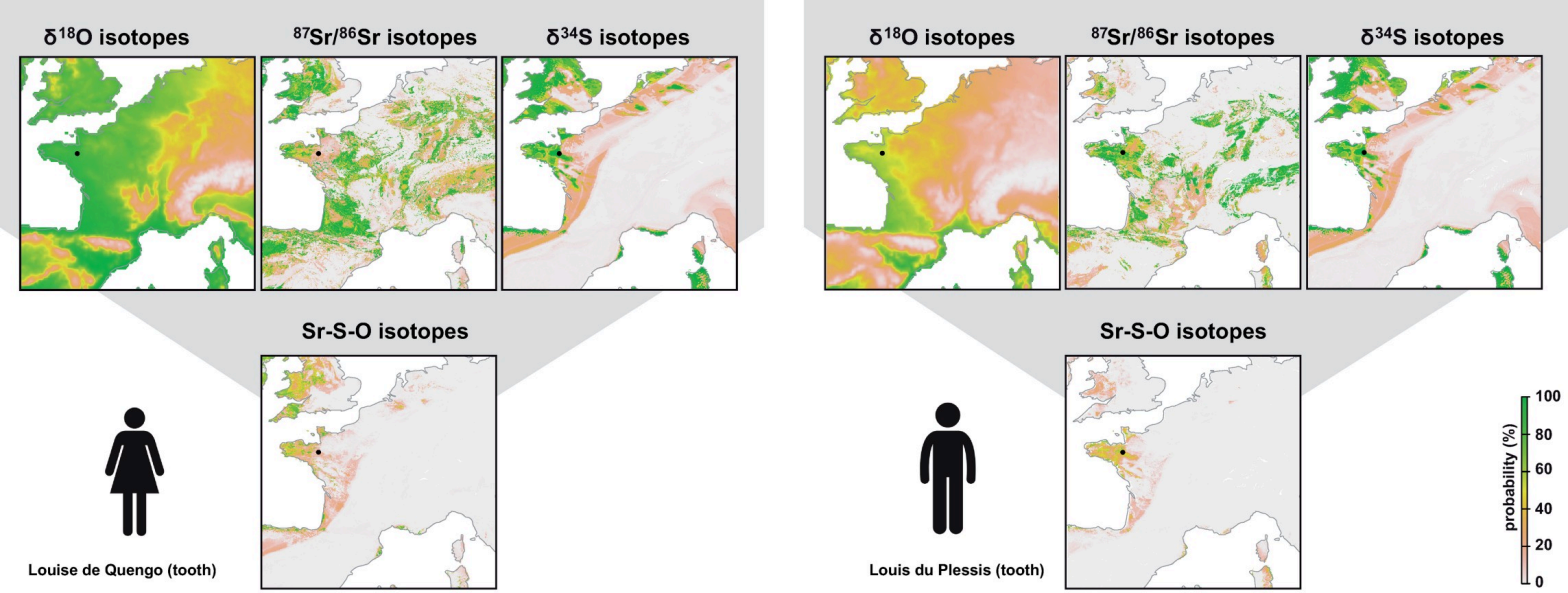
Maps showing the probability of origin of Louise de Quengo and Louis de Plessis based on single and triple isotopes geographic assignments. To generate these figures the script is available in S1 and S2 Scripts.

To facilitate discussion and visualization, we displayed all the isotopic assignments for one individual (Fig 3). We chose the dog because unlike cattle which could be imported from regional production sites, the dog was likely local to the convent or to the city of Rennes. We use this local individual to compare the geographic assignments using each isotopic system and their combination. At the individual level, geographic assignment using δ^18^O in the tooth displayed broad regions of potential origin covering most of Western Europe except mountainous zones (Fig 3). ⁸⁷Sr/⁸⁶Sr geographic assignment showed discrete region of origin and higher precision focusing on mountainous regions of Western Europe (Fig 3). The δ^34^S value in the tooth limit potential origin to coastal region of western Europe, including the United Kingdom (Fig 3). Dual and triple isotope assignments largely improved the precision with triple isotopes assignments restricting high probability to central Brittany (Fig 3). Similar observations are made on the triple isotopes assignments of other animals and humans (Figs 4 and 5).

## 4. Discussion

### 4.1 Sulfur isoscape

As observed in previous studies [38, 62], the δ^34^S values in human and animal collagen increases closer to the coast when sea salt aerosol deposition increases through precipitation (Fig 1C). This increase could be related to two factors: 1) Food systems near the coast receive a higher proportion of isotopically heavy marine sulfates and/or 2) humans living near the coast eat more isotopically heavy seafood. We argue that the first hypothesis is more likely as both animals and humans show the same pattern, and animals (particularly herbivores) do not eat seafood. We notice that the relationship between sea salt aerosol deposition and distance to the coast with the predicted δ^34^S values is not linear, confirming previous studies [62]. Close to the coast (<100km) when sea salt aerosol deposition rate is high, the predicted δ^34^S values are relatively constant. At distances greater than 150 km when sea salt aerosol deposition rate decreases below 2 g.m^-2^.yr^-1^, δ^34^S values in human collagen drop rapidly [29]. At distances greater than 200km from the coast and when sea salt aerosol depositions are low (<2 g.m^-2^.yr^-1^), δ^34^S values in human collagen are not influenced by sea salt deposition rate, and other factors control δ^34^S values.

Dust aerosols also exert a strong control on δ^34^S variations with increasing δ^34^S values for higher dust aerosol deposition (Fig 1D). In Europe, dust aerosols originate primarily from the Sahara desert [63]. Sahara dust contains significant amount of sulfur from gypsum sourced from marine evaporite basins [64]. These sediments have high δ^34^S values ranging from 12–16 ‰ [65]. The high deposition of Sahara dust in southern Europe contributes to the high δ^34^S values in these regions.

Positive Bouguer anomalies (>100mGal) are associated with higher δ^34^S values, whereas low and negative Bouguer anomalies are associated with low δ^34^S values (Fig 1E). Negative Bouguer anomaly across Europe include all active mountain zones and their associated sedimentary wedges, the Scandinavian Shield, and the interior of the Iberian Peninsula. All these geological settings tend to expose older igneous or metamorphic rock units and terranes of late Phanerozoic to Precambrian ages. These rocks units are likely richer in isotopically-light sulfides than other terranes explaining their lower δ^34^S values [66]. Conversely positive Bouguer anomalies across Europe are mostly located along the coastlines particularly on the western side where sea salt aerosol deposition is high, and this geographic distribution could fortuitously explain their higher δ^34^S values. Except for Bouguer anomalies, no geological or soil variables (i.e., lithology, age, clay content or soil pH) were selected by the *VSURF* algorithm. This lack of geological controls is a bit surprising because δ^34^S values are known to be influenced by bedrock geology [29]. For example, in areas with bedrock containing high sulfide content (e.g., marine shale), ecosystems usually display low or negative δ^34^S values [31, 37]. The compiled dataset encompasses only a few sites with negative δ^34^S values and the predicted δ^34^S values at these sites overestimate the observed δ^34^S by several per mile (Fig 1A). At these sites, sulfur sources are probably dominated by geogenic sulfides with low δ^34^S but this control is not well-represented in our current model. For example, eastern England and northeastern France have a similar geology composed primarily of Jurassic formation rich in organic black shales. These rocks were deposited in the European epicontinental shelf of the Tethys Ocean, often under anoxic conditions, and are rich in sulfides with low δ^34^S values [67]. Humans recovered from archaeological sites in these regions show very low δ^34^S values [45, 68, 69]. Similarly, freshwater animals from rivers draining these rock units also show low δ^34^S values [45]. Compiling additional sites from sulfide-rich regions could help improve the representation of geogenic controls in δ^34^S isoscapes. Another reason for the lack of geological controls on δ^34^S values is that we trained the model using δ^34^S values from human/animal tissues. Humans incorporate sulfur from multiple local sources mixing sulfur from different locations. This is not a problem for assessing the impact of climatic variables (e.g., aerosols) which usually vary across broad spatial gradients but it becomes more problematic to test the influence of geological controls that vary more discretely and locally on the landscape.

The δ^34^S isoscape developed from this random forest regression approach shows strong patterns that are promising for provenancing (Fig 2). δ^34^S values are high in coastal regions but are particularly high in northwestern Europe (e.g., Scotland). Northwestern Europe receives the highest rate of sea salt aerosols of Europe because the high wind in the North Atlantic leads to high sea salt entrainments that are deposited through high mean annual precipitation in these regions [63]. Sulfates from these aerosols are dominated by marine sea salt with high δ^34^S values, but sulfates from DMS with lower δ^34^S values also contribute to sulfate deposition [70]. The circum-Mediterranean region has moderately high δ^34^S values influenced by moderate marine sea salt deposition and high Sahara dust aerosols [63], with locally high values (e.g., Crete) associated with high sea surface winds. The lowest δ^34^S values are found inland, including within the Paris basin, Germany, Poland and Eastern Europe, where geogenic controls dominate the local S cycle in ecosystems.

### 4.2 Single vs. dual vs. triple isotopes assignments

#### 4.2.1 Dog individual

We find that the dog individual showed high probability to originate from the region of Rennes for single δ^34^S, ^87^Sr/^86^Sr and δ^18^O assignments (Fig 3). In this study, geographic assignment using δ^34^S consistently outperformed those conducted using δ^18^O values and show similar precision to those performed with ^87^Sr/^86^Sr. This is promising, considering that the uncertainty of the δ^34^S isoscape was conservatively set at 3‰. This value likely overestimates uncertainty in most regions. Reducing the uncertainty of the δ^34^S isoscape could further increase the precision of geographic assignments. Improving the isoscape will require higher sampling density across Western Europe and novel algorithms to incorporate spatial uncertainty within the random forest regression framework (e.g., quantile random forest).

However, we must also keep in mind that the high performance of δ^34^S geographic assignments is strongly dependent on the location of study. In this particular study, δ^34^S perform well at reducing the potential area of origin because the archaeological site is located close to the coast. Coastal regions have diagnostic δ^34^S values with rapid spatial variations over a few hundreds of kilometers. Sites located more inland would have less diagnostic δ^34^S values. For example, archaeological site within eastern Europe can hardly be distinguished using δ^34^S values (Fig 2). At those sites other isotopic systems, such as strontium or oxygen, might be more diagnostic due to specific geological or climatological conditions.

As illustrated for the dog individual, when used independently, single isotope assignments show a broad range of potential origin and low precision but when used in combination these isotopes can provide precise provenance (Fig 3). The δ^18^O values of the dog are compatible with most of France (Fig 3). δ^34^S values of the dog are compatible with most of coastal western Europe (Fig 3). ^87^Sr/^86^Sr ratios of the dog are compatible with many radiogenic area of France (e.g., Massif Central, Alps, Aquitaine) (Fig 3). However, these single isotope assignments are not sufficient to confirm this individual is local from Brittany. When combining these isotopic systems in dual and triple isotopic assignments, we find that the region of origin narrows down considerably (Fig 3). Triple isotopic assignments show highest probability of origin in central Brittany and around Rennes, with most of the remaining areas across Europe removed (Fig 3). This underlines the interest of quantitatively combining isotopic systems because each isotope has its own strength and limitation in terms of constraining provenance and their potential depends on the site studied. At this site, the geology and coastal setting provide strongly diagnostic isotopic signatures facilitating the recognition of local individuals. Using triple isotope geographic assignments should strongly help to distinguish individuals that are local to Brittany against individuals that could come from other regions of Europe. For example, mass graves discovered in the Dominican Convent of Rennes contain dozens of unidentified individuals that could be related to specific events using multi-isotope assignments (see [42])

#### 4.2.2 Animals

Based on δ^34^S assignments, all animals show a high probability of local geographic origin (Fig 4). Based on ^87^Sr/^86^Sr geographic assignment, these local animals are all compatible with the expected range for Brittany. Based on δ^18^O geographic assignment, the δ^18^O are also all compatible with locations around Rennes. However, the M1 sample from a cow show comparatively low probability of origin in Brittany (Fig 4). M1 teeth are formed during the first year of life, and the suckling likely impacted the δ^18^O values of the cow. We also did not sample the whole height of the crown, and the molars of herbivore teeth are much bigger than carnivores and human ones. These large teeth can record seasonality. Dual isotope assignments show very high probability of local origin for all individuals. Triple isotope assignments are even more convincing, showing that many of these animals could only have come from Brittany (Fig 4). These results validate our approach and suggest that triple isotope assignments can provide precise information about the region of origin of individuals (Fig 4).

#### 4.2.3 Local humans with known identity

The single, dual and triple geographic assignments for Louise de Quengo and Louis du Plessis show patterns very similar to those observed for the dog individual (Fig 5). Each single isotope assignment is not diagnostic enough to infer an origin from Brittany. However, when combining the three isotopes, both individuals display a very high probability of origin in Brittany validating historical data. The tooth of Louise de Quengo shows a high probability of origin in central Brittany though her isotopic values are also compatible with the local Rennes range (Fig 5). The tooth of Louis du Plessis has a dual δ^34^S -^87^Sr/^86^Sr assignment compatible with an origin around Rennes. His δ^18^O value is a bit high compared to other individuals from Brittany. Diagenesis of the tooth is unlikely to explain the high δ^18^O value because Louis du Plessis’ body was partially mummified and buried in a lead coffin [54]. Warmer than usual climate is another possibility for explaining high δ^18^O values in human teeth. However, Louis du Plessis was born around 1610 living his early life during the coldest part of the Little Ice Age in Europe. We suggest that the high δ^18^O value on this premolar could reflect late weaning. The δ^18^O value in breastmilk is more enriched in ^18^O than drinking water and can overprint local δ^18^O value in human premolars [71].

### 4.3 Implications and future improvements

We demonstrated that triple δ^34^S -^87^Sr/^86^Sr—δ^18^O geographic assignment can considerably increase the precision of geographic assignment in comparison with single isotope assignment. In this particular study, δ^18^O assignments had the worst precision. This low performance of δ^18^O assignments is associated with the high uncertainty of δ^18^O isoscape in teeth. Analyzing a larger dataset of δ^18^O values in the teeth of individuals with known origin will be critical to further improve the potential precision of δ^18^O assignments. However, as observed in this study and others, δ^18^O values show large variability within teeth and between individuals at a given site. Fractionation associated with specific diet, physiology, or tissue can superimpose δ^18^O variations driven by geography [15]. While δ^18^O values are the most used isotopic system in provenance studies in archaeology, it is often not the best tool to assess origin of individuals. In this study, δ^34^S values combined with ^87^Sr/^86^Sr are highly diagnostic to identify individuals from Brittany and even potentially identifying origin within Brittany. δ^34^S values have been primarily used to reconstruct dietary choices (e.g., fish consumption) [44]. However, this study demonstrates that their potential for provenancing have been strongly overlooked in archaeology.

## 5. Conclusion

While our study is not the first to combine multiple isotopic systems in archaeological reconstruction, it is the first study that interprets triple S-O-Sr isotope data in a quantitative probabilistic framework. Such advance was allowed by the development of a novel δ^34^S isoscape predicting δ^34^S in archaeological human teeth across western Europe. We used this new isoscape for single isotope geographic assignment, but also combined it with Sr and O isotope data to produce triple isotope probabilistic geographic assignments. Our study represents a major advance in archaeology and paleoecology as it lays the framework to use dual and triple isotope systems in quantitative probabilistic approaches. This method paves the way for more accurate and precise provenance studies in archaeology.

## Supporting information

S1 Script (R)

S2 Script (TXT)

S1 Dataset (XLSX)

S2 Dataset (CSV)
